# moBRCA-net: a breast cancer subtype classification framework based on multi-omics attention neural networks

**DOI:** 10.1186/s12859-023-05273-5

**Published:** 2023-04-26

**Authors:** Joung Min Choi, Heejoon Chae

**Affiliations:** 1 Department of Computer Science, Virginia Tech, Blacksburg, USA; 2 Division of Computer Science, Sookmyung Women’s University, Seoul, Republic of Korea

**Keywords:** Attention, Breast cancer subtype classification, Deep learning-based framework, Multi-omics, Neural network

## Abstract

**Background:**

Breast cancer is a highly heterogeneous disease that comprises multiple biological components. Owing its diversity, patients have different prognostic outcomes; hence, early diagnosis and accurate subtype prediction are critical for treatment. Standardized breast cancer subtyping systems, mainly based on single-omics datasets, have been developed to ensure proper treatment in a systematic manner. Recently, multi-omics data integration has attracted attention to provide a comprehensive view of patients but poses a challenge due to the high dimensionality. In recent years, deep learning-based approaches have been proposed, but they still present several limitations.

**Results:**

In this study, we describe moBRCA-net, an interpretable deep learning-based breast cancer subtype classification framework that uses multi-omics datasets. Three omics datasets comprising gene expression, DNA methylation and microRNA expression data were integrated while considering the biological relationships among them, and a self-attention module was applied to each omics dataset to capture the relative importance of each feature. The features were then transformed to new representations considering the respective learned importance, allowing moBRCA-net to predict the subtype.

**Conclusions:**

Experimental results confirmed that moBRCA-net has a significantly enhanced performance compared with other methods, and the effectiveness of multi-omics integration and omics-level attention were identified. moBRCA-net is publicly available at https://github.com/cbi-bioinfo/moBRCA-net.

**Supplementary Information:**

The online version contains supplementary material available at 10.1186/s12859-023-05273-5.

## Background

Breast cancer is one of the most frequently diagnosed cancers and is the second leading cause of death among women worldwide [1]. Breast cancer has been characterized as a heterogeneous disease composed of diverse biological factors, including genetic components and epigenetic modifications, that differentially impact on the prognostic and clinical outcomes of patients [2]. This diversity represents a challenge for the development of systems to classify breast cancer, which are clinically useful with respect to prognosis and prediction [3]. The development of microarrays has led to a new paradigm in deciphering cancer heterogeneity, allowing to divide breast cancers into subtypes based on their molecular profiles [4]. Pioneer studies by Sorlie et al. revealed the molecular properties of human breast cancer and proposed a patient stratification system according to the clinical outcomes of their cancer subtype, classifying breast cancer into five intrinsic subtypes: luminal A, luminal B, HER2 overexpression, basal-like, and normal-like cancers [5]; which have been further supported by several other studies based on gene expression profiling. Moreover, Parker et al. presented the PAM50 model that was based on the signature genes related to the hormone receptor, proliferation, and myoepithelial and basal features [6], which has become a standardized subtyping system that can be widely applied in the clinical setting. Accurate prediction of the breast cancer subtype based on those categories has become crucial to cancer prognosis, which helps for therapeutic decision making and to ultimately improve patient outcomes [7].

In recent years, several classification frameworks to predict the molecular subtypes of breast cancer have been reported. For example, Rhee et al. presented a hybrid approach integrating graph convolutional network and relation network using gene expression profiles and protein–protein interaction networks [8]. Gao et al. [9] designed DeepCC, an algorithm of enrichment score calculation for each gene expression profile of cancer samples based on the selected gene sets, which classified the breast cancer subtypes by implementing the fully connected neural network model using those scores. Beykikhoshk el al. [10] proposed a framework to classify the gene expression signatures of luminal A and luminal B breast cancer subtypes by calculating the personalized biomarker scores based on the attention mechanism. Lee et al. [11] developed a cancer subtype prediction framework using pathways by modeling a multi-attention-based graph convolutional network, which presented a stable performance for classifying breast cancer subtypes. Zhezhou et al. [12] performed differential expression analysis on biologically important genes from gene regulatory networks and constructed a machine learning-based binary classification model for each breast cancer subtype with the obtained differentially expressed genes.

However, classification approaches based solely on single-omics datasets only provide information on a single molecular level. These approaches fail to provide a comprehensive view of the biological processes involved in breast cancer and cannot capture correlations between molecules from different layers [13]. To overcome this limitation, efforts for collecting multi-omics datasets from each subject have been made [14, 15], and integrative approaches combining multi-omics data have been proposed to discover the coherent biological signature, and improve the prognostic and predictive accuracy of disease phenotypes [16]. Various studies have shown that combining multiple omics datasets yield better understanding and more accurate prediction to clinical outcomes, thereby proving the importance of integrating multi-omics datasets over single-omics [17, 18]. Indeed, this approach was adopted to breast cancer studies. For example, List et al. constructed random forest models to classify breast cancer subtypes using both gene expression and DNA methylation datasets, and evaluated the performance of the model when was trained based multi-omics in comparison with that of single-omics-based classification [19]. Further, an approach to integrate mRNA, methylation, and copy number variation (CNV) data using a multiple kernel learning model has been proposed for breast cancer subtype prediction [20]. However, due to the high dimensionality of the multi-omics data, the classification performance of those models did not show significant improvement compared with that of single-omics-based approaches.

Recently, deep learning-based multi-omics data integration has shown robust performance in several applications, including patient clustering, survival analysis [21], and biomedical classification. MOGONET [22] jointly trained the omics-specific learning and cross-omics correlation learning for several classification applications including the tumor grade classification, and MOMA [23] presented a multi-task attention learning algorithm for disease prediction based on two omics data integration. Supported by those results, a similar approach was also developed for breast cancer subtype classification. HI-DFNForest [24] employed neural network-based deep forest model to integrate DNA methylation, microRNA, and gene expression datasets. In turn, DeepMO [25] applied fully-connected layers to each omics (DNA methylation, mRNA, and CNV data) and concatenated those subnetworks for final subtype prediction. These neural network-based frameworks showed good subtyping performance; however, these breast cancer subtype classification models are still limited by the difficulty to explain how they are trained, to define which features from each omics dataset play a key predictive role, and whether those models assign more weights to these features. To solve these issues caused by the black-box nature of neural networks, self-attention mechanisms [26] have been presented, which allow neural networks to focus on important regions of input data for better prediction by training the model to learn the relative importance of each feature of the input data. This novel approach has been widely employed in single-omics-based studies, including for breast cancer subtype classification [10, 11]. However, due to the high costs of computing a large number of features and the significant complexity of omics integration, multi-omics-based methods have not been easily adopted.

In the present study, we propose moBRCA-net, an omics-level attention-based breast cancer subtype classification framework that uses multi-omics datasets. Dataset integration was performed based on feature-selection modules that consider the biological relationship between the omics datasets (gene expression, DNA methylation, and microRNA expression). Moreover, for omics-level feature importance learning, a self-attention module was applied for each omics feature, and each feature was then transformed to the new representation incorporating its relative importance for the classification task. The representation of each omics dataset was concatenated and delivered to the fully connected layers to predict the breast cancer subtype of each patient.

## Methods

**Fig. 1:**
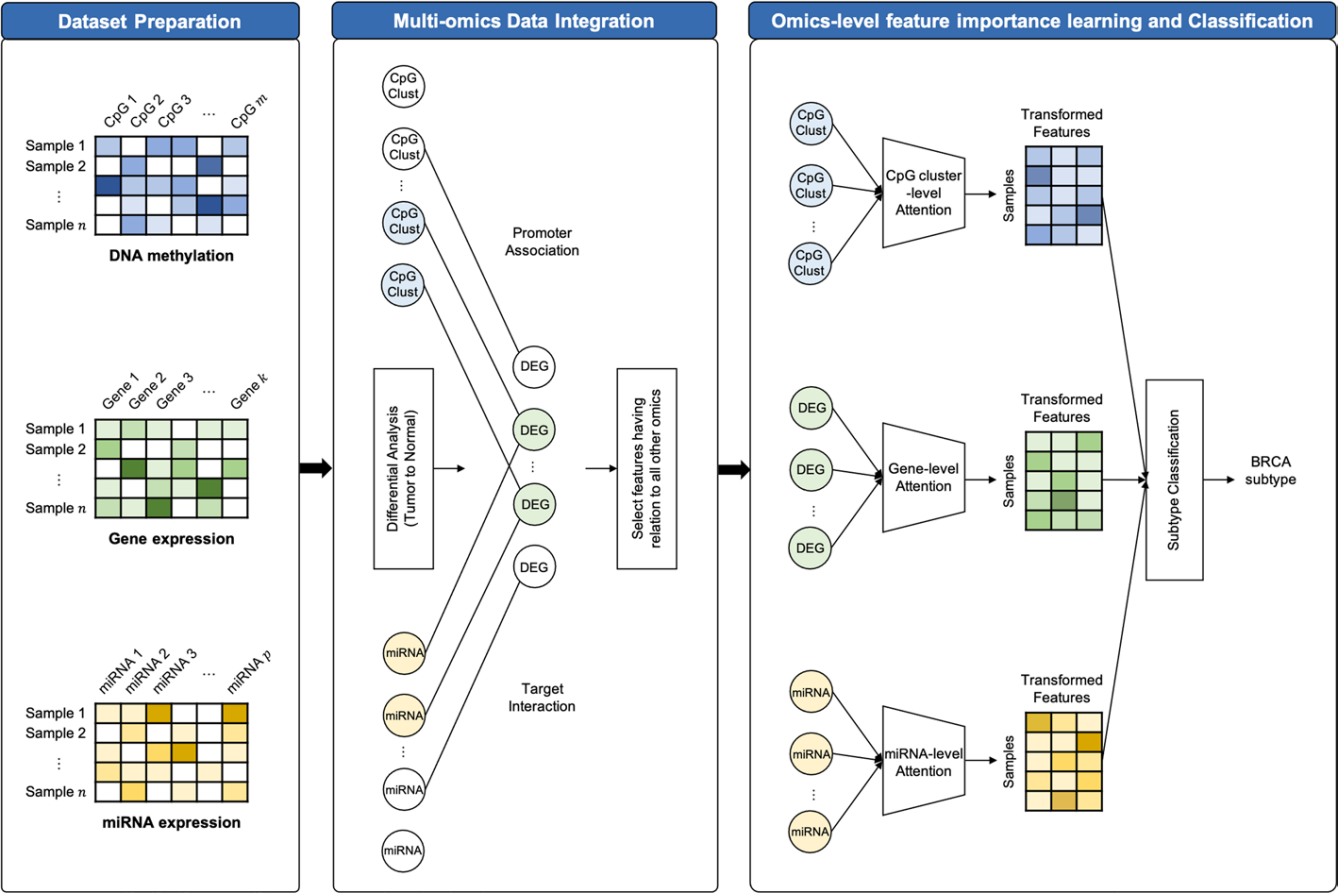
Illustration of the proposed BRCA subtype classification model based on a multi-omics data integration

Our moBRCA-net framework consists of four modules: preprocessing, multi-omics data integration, omics-level feature importance learning, and classification. The workflow of our model is shown in Fig. 1.

### Dataset

The breast cancer (BRCA) cohort datasets were collected from The Cancer Genome Atlas (TCGA) [14]. Three types of omics datasets—gene expression, DNA methylation, and microRNA expression—were obtained. Patients who did not have all three omics data available were excluded. Breast cancer subtype information for each TCGA BRCA sample was retrieved from PAM50 [6]. Overall, a total of 1059 samples were divided into five subtypes, as shown in Table 1.

**Table 1:** Number of samples for each breast cancer subtype

Breast cancer subtype	Number of samples
Luminal A	556
Luminal B	200
HER2-enriched	182
Basal-like	81
Normal-like	40

### Preprocessing

For gene expression and microRNA expression data, we first removed genes and microRNAs for which read counts were not available for all samples. After calculating size factors, the read counts were normalized by library size and were log-transformed using DESeq2 [27]. For DNA methylation data, both DNA methylome datasets measured by Illumina Human Infinium 450 K and 27 K platforms were used, with common features of both datasets being used for further analysis. To eliminate the bias caused by a high frequency of missing values during model training, median imputation was performed, in which CpG sites with missing values for all samples were removed, resulting in the retention of 20,400 genes, 19,977 CpGs, and 1597 microRNAs.

### Multi-omics data integration

To prevent a possibility of overfitting and expensive computational costs to train the neural network model, we constructed a feature selection module to integrate the multi-omics datasets. First, the genes remaining after preprocessing were evaluated concerning their informativity as a breast cancer signature by performing differential analysis that compared them to matched normal samples using DESeq2. Genes with an absolute value of log (fold change) greater than 2 and an adjusted *p*-value less than 0.01 was considered as differentially expressed genes (DEGs). We constructed the gene set composed of 1000 DEGs with the highest log(fold change) values and the lowest adjusted *p*-value.

To integrate the multiple omics datasets based on their biological relationship, we identified the other omics features related to the selected DEGs. Studies have shown that promoter-associated CpGs play important roles in gene silencing, genomic imprinting, and cancerigenesis [28–30]. After preprocessing, CpGs within 2 kb of the promoter regions of each DEG were grouped to form a cluster (hereafter referred to as CpG cluster), where the average of the beta values were calculated. DEGs without matched CpGs in the perprocessed dataset were filtered out to focus on features related to other omics. In addition, microRNAs control the function of their target mRNAs by downregulating the expression of their targets [31]; thus, they have been recognized as drivers of diverse disease conditions including cancer [32]. microRNAs showing target interaction with the identified DEGs were selected based on the TargetScan database [33].

### Omics-level feature importance learning

To learn the features playing a key role for classifying the breast cancer subtypes and better understand the relative importance of those features, we implemented the omics-level self-attention module into our model. Given a set of original input data $x \in {{\textbf {R}}}^n$, where *n* represents the dimension of the input data, we defined the *k*-dimensional embedding vector $e_i$ for each feature $i \in \{1:n\}$ using random vectors and represented $x_i$ to ${\hat{x}}_i$ via multiplication as follows [10]:

1 $$\begin{aligned} \hat{x}_i = f_e(e_i,x_i) = e_i x_i \end{aligned}$$

The original input *x* was transformed to a new representation $\hat{x}$ with the embedding vectors $\hat{x} \in {{\textbf {R}}}^{n \times k}$. To learn the level of importance for each feature to predict breast cancer subtype, each feature $\hat{x}_i$ was assigned an attention score $\alpha _i$ as follows:

2 $$\begin{aligned} \bar{x}_i&= tanh(W_{FC} \hat{x}_i + b) \end{aligned}$$

3 $$\begin{aligned} s_i&= W_{h2} tanh(W_{h1} \hat{x}_i + b) \end{aligned}$$

4 $$\begin{aligned} \alpha _i&= {{\exp (s_i)} \over {\sum _{j=1}^n \exp (s_j)}} \end{aligned}$$

5 $$\begin{aligned} c_i&= \sum _{i=1}^n \alpha _i \bar{x}_i, \end{aligned}$$

where $W_{FC}$, $W_{h1}$, and $W_{h2}$ are the weights and *b* is a bias term. $s_i$ is the attention score that represents the importance of each feature $\hat{x}_i$, which was converted to a normalized weight $\alpha _i$ by applying the softmax function. Based on the calculated values, $\hat{x}_i$ was transformed to a dense feature representation $c_i$ by the weighted sum of the encoded feature vectors $\bar{x}_i$ and their normalized attention scores $\alpha _i$. The self-attention module was applied to each omics dataset, and the transformed feature representation of each module was concatenated and delivered to the subtype classification module.

### Subtype classification

The classification module was constructed with two fully connected layers followed by the softmax function layer to achieve the final breast cancer subtype classification. moBRCA-net was trained to minimize the cross-entropy loss, defined as follows:

6 $$\begin{aligned} \mathcal {L} = -\sum _{i=1}^{C} y_i \cdot \log (\hat{y}_i), \end{aligned}$$

where *C* represents the number of breast cancer subtypes, and *y* ($\hat{y}$) is the true (model predicted, respectively) subtype probability distribution. To prevent overfitting, dropout [34] was applied, and L2 regularization was also added to the loss function. We used the adaptive moment estimation (Adam) optimization algorithm [35] for training.

For optimization, we randomly selected 70% of the samples as a training dataset and the remaining 30% of the samples as test dataset. The experiment was repeated three times for each hyperparameter combination, and the architecture showing the best average accuracy result was set as our moBRCA-net model. The dimensions of the embedding vector *k* and encoding vector $\bar{x}$ were set as 128 and 64, respectively, and the dimension of the attention vector *s* and $\alpha$ were the same as the number of features. For the classification module, two fully connected layers with 200 and 5 hidden nodes were used with the ELU activation function. The dropout rate was set to 0.7 for both training the omics-level attention and the classification module. The learning rate and training epoch were set as 0.01 and 5000, respectively. The optimization results from the experiments with different parameters are shown in Additional file 1: S1. Performance results for optimizing the number of selected features during the multi-omics integration are also shown. Our proposed model was built using Tensorflow library (Version 1.8.0).

## Results

### Evaluation of moBRCA-net performance

To evaluate the ability of moBRCA-net to classifying breast cancer subtypes, we compared its performance with that of widely-used machine learning(ML)-based classifiers: Support vector machine (SVM) [36], Random Forest (RF) [37], Logistic Regression (LR) [38], and Naive Bayes (NB) [39], which were implemented using the Scikit-learn package [40]. Following the same optimization procedure used for moBRCA-net, the baseline methods were optimized based on the TCGA-BRCA dataset, where the training and testing datasets were randomly split to the ratio of 7:3. Grid search was adopted for the model tuning. For each combination of hyperparameters, the experiment was repeated five times, and the parameters showing the highest average accuracy for the testing dataset were selected (Additional file 1: S2). The optimized hyperparameter settings for each classifier are as follows: SVM (kernel = rbf, C = $2^3$, gamma = $2^{-7}$), RF (criterion = gini, estimators = 100, min_samples_leaf = 3), LR (max_iter = 100, C = 2). Tenfold cross validation was performed, where the training dataset of each fold was used for the multi-omics data integration and training the model, and testing dataset was only used for measuring the performance, not being included to any other steps. For comparison methods, the same multi-omics features selected from moBRCA-net were used, and the accuracy, the weighted F1-score, and MCC were adopted as evaluation metrics. To show the significance of performance differences between the comparison methods and our proposed method, the Wilcoxon signed rank test was performed.

**Fig. 2:**
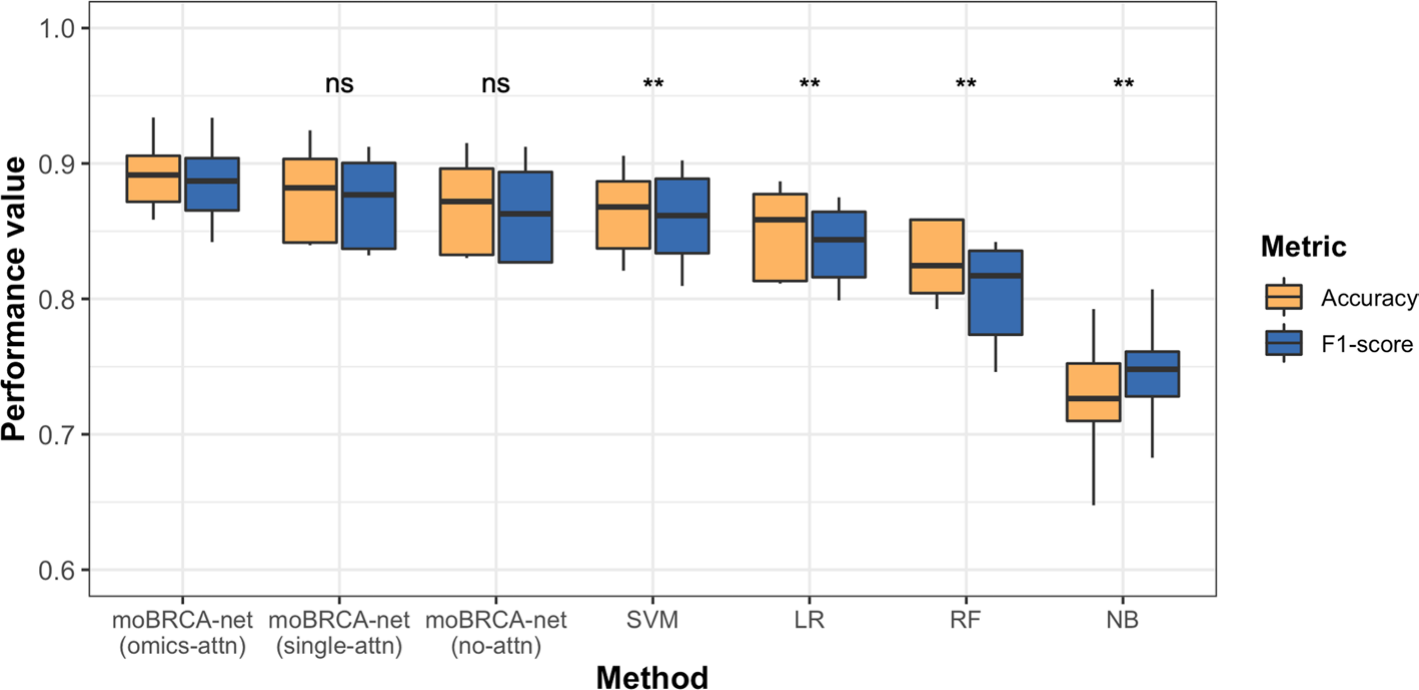
Performance comparison of machine learning classification methods with moBRCA-net and its variants by tenfold cross-validation. For significance testing, Wilcoxon signed rank test was performed using the performance results from moBRCA-net (omics-net) and results from each baseline method. Notations: *ns* Not significant; **p-value $< 0.01$

Overall, moBRCA-net (denoted as ’moBRCA-net (omics-attn)’) significantly outperformed the other ML-based classifiers with an average accuracy of 0.891, F1-score of 0.887, and MCC of 0.831. SVM showed the second-best performance with 0.866, 0.861, and 0.808 for the average accuracy, F1-score, and MCC respectively (Fig. 2, Additional file 1: S3). Regarding the highest performance obtained, our proposed model achieved an accuracy and F1-score of 0.934 and 0.934, respectively. Subtype-wise performance results for our moBRCA-net (omics-attn) is shown in Additional file 1: S4. Next, we compared the performance of our model with that of the state-of-the-art cancer subtype classifier models, HI-DFNForest and MOGONET. We could not reproduce the experiments using HI-DFNForest since the authors did not make available its implementation code. Nevertheless, we carefully performed the same evaluation based on fivefold cross validation following the same experimental setting described in the HI-DFNForest report. moBRCA-net showed a higher average accuracy of 0.897 as compared with HI-DFNForest, which achieved an average accuracy of 0.846. MOGONET was also compared following the same experimental setting in the paper. The dataset was randomly split to the ratio of 7:3 as a training and testing dataset, and the method was evaluated on five different randomly generated splits. From the results, moBRCA-net achieved the higher average accuracy of 0.867 and the weighted F1-score of 0.864, outperforming the MOGONET having the average accuracy of 0.815 and the weighted F1-score of 0.821.

Our moBRCA-net has shown the performance improvement compared to the baseline classifiers, but still the data imbalance issue could impact the prediction performance in a subtype-specific fashion, where there is a large difference between the number of samples for each subtype. We performed an additional experiment to investigate a strategy to alleviate this concern while training a classifier. For better generalization of moBRCA-net, we adopted a data augmentation based on the deep generative model to enlarge the training dataset size. Several recent papers have shown that conditional variational autoencoder (CVAE)-based data generation for certain minority classes in the imbalanced dataset improved the classification performance in various domain tasks such as respiratory disease classification [41], temporal pattern prediction based on electronic health records [42], and prediction of chemical structure based on the chemical properties [43]. We constructed a conditional variational autoencoder (CVAE) composed of two-layered encoder and decoder, which estimates the conditional distribution with latent variables and data, and generates samples for specified breast cancer subtype. For each fold dataset in tenfold cross validation, CVAE was optimized based on the training dataset and generated the samples for each subtype to match the same number of samples for“Luminal A” subtype having the largest number of samples. Those generated samples were added while training moBRCA-net, and the performance was measured for the testing dataset. From the results (Additional file 1: S5), the overall performance of moBRCA-net as well as the subtype-wise performance slightly improved compared to the model trained without the generated dataset. These results support that data augmentation strategy could help to alleviate the impact from the imbalanced dataset while training our model. The implementation code for our CVAE is provided in our GitHub repository.

### Effectiveness of each module in moBRCA-net

Next, we investigated the performance improvement of moBRCA-net by the introduction of the omics-level attention modules for feature importance learning. We implemented two variants of moBRCA-net, in one a single-attention module was applied to all features at once, and in the other the attention module was removed to directly classify the breast cancer subtypes, which were denoted as ’moBRCA-net (single-attn)’ and ’moBRCA-net (no-attn)’, respectively. tenfold cross-validation was performed to measure performance. Without the attention module, the classification performance was comparable to that of the SVM, showing an average F1-score of 0.864 (Fig. 2, Additional file 1: S3). However, when the single attention module was applied, moBRCA-net average F1-score was improved to 0.872. Interestingly, by applying omics-level attention, the performance significantly increased to 0.887. These results demonstrate the effectiveness of the attention modules to learn the importance of the features of each omics dataset and train the model to focus more on those that play a key role in breast cancer subtype prediction. These experiments also suggest that applying attention at the proper level has an impact on learning the features and modeling the classification module.

**Fig. 3:**
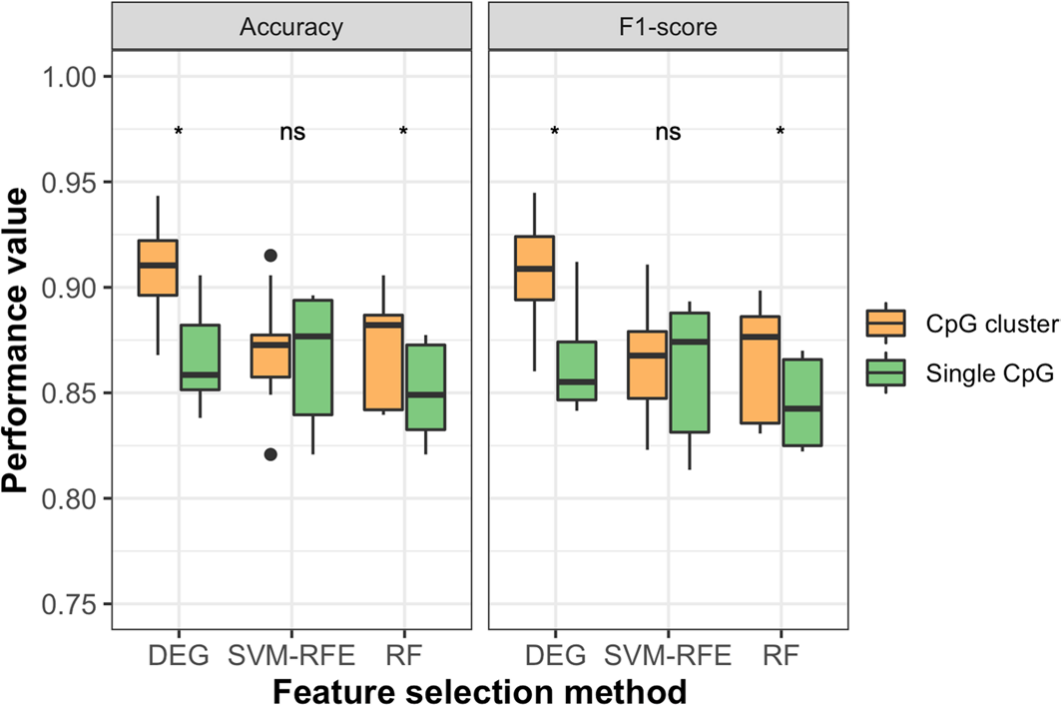
Subtype classification performance integrating multiple omics datasets with different feature selection methods based on tenfold cross validation. For significance testing, Wilcoxon signed rank test was performed using the CpG-cluster-based performance results and results based on the single-CpG for each feature selection method. Notations: *ns* Not significant; *p-value $< 0.05$

Before constructing the neural network model, we integrated multi-omics based on the feature selection and grouping of related CpGs to create CpG clusters. Thus, we evaluated next the impact of each of these steps on breast cancer subtype prediction. First, we evaluated the classification performance by changing the feature-selection method in moBRCA-net and compared it with that of two widely used feature selection methods: support vector machine-recursive feature elimination (SVM-RFE) [44] and RF. The same number of genes was selected as the number of DEGs in moBRCA-net based on the classification task for predicting cancer and normal samples. Next, related CpG clusters and microRNAs were extracted based on the selected genes. From the tenfold cross validation results (Fig. 3, Additional file 1: S6), we could observe that DEG-based feature selection achieved the highest performance in terms of both accuracy and F1-score, showing that the DEG-based method detects more discriminative features for identifying cancer and normal samples, which may include the representative genes for each subtype.

Furthermore, we investigated the impact of the CpG clusters by comparing the classification performance of moBRCA-net based on single CpG-based multi-omics integration using different feature selection methods (Fig. 3). Interestingly, when utilizing CpG clusters, the average classification performance significantly improved for DEG-based method (from F1-score of 0.864 to 0.908) and RF (from 0.845 to 0.866). SVM-RFE showed a slight performance increase (from 0.86 to 0.866). We also observed that CpG cluster-based approaches achieved the best accuracy and F1-score compared with single-CpG approaches for all cases using different feature selection methods. We assumed that CpGs located in regions relatively close to the promoter may share a similar methylation status, which could represent the methylation patterns related to breast cancers, consequently leading to performance improvement for subtype prediction.

**Table 2:** Average classification performance for breast cancer subtype classification using different omics datasets

	Multi-omics				Single omics		
	Gene + methyl + microRNA	Gene + methyl	Gene + microRNA	Methyl + microRNA	Gene	Methyl	MicroRNA
Accuracy	0.909	0.865	0.889	0.820	0.863	0.817	0.85
F1-score	0.908	0.857	0.886	0.807	0.852	0.806	0.842
*p*-value	–	< 0.05	< 0.05	< 0.01	< 0.05	< 0.01	< 0.01

Wilcoxon signed rank test was performed using the performance results using all three omics dataset and results from each combination of different omics dataset

**Fig. 4:**
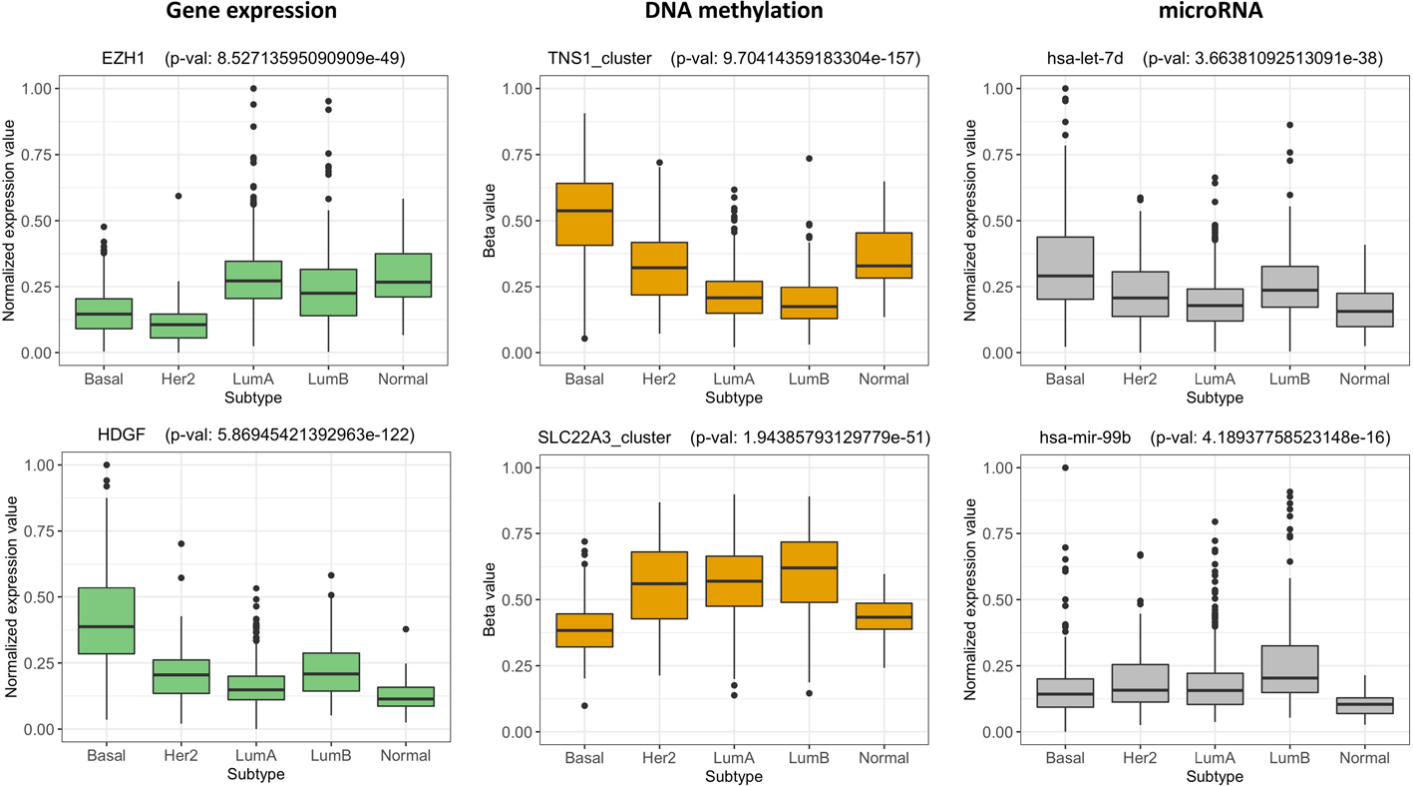
Normalized values of the features from each omics dataset showing the top average attention scores

### Breast cancer subtype prediction improvement by multi-omics integration

To validate whether utilizing multi-omics datasets could effectively improve the classification of breast cancer subtypes, we compared the performance of moBRCA-net when using different combinations of multi-omics datasets and a single-omics dataset. We denoted gene expression as ’gene’, DNA methylation as ’methyl’, and microRNA as ’microRNA’. In this experiment, moBRCA-net showed a relatively higher performance when trained based on multi-omics compared with single-omics data (Table 2). Multi-omics dataset integrating gene expression, DNA methylation patterns, and microRNA expression achieved the highest average accuracy of 0.909 and F1-score of 0.908. Breast cancer subtype classification performance also slightly increased when two omics datasets were used, but incorporation of three omics datasets still achieved a much higher classification improvement. These results indicate that multi-omics data provides more comprehensive information to distinguish breast cancer subtypes.

### Interpretation of omics-level attention for breast cancer subtype classification

To understand how omics-level attention helped improve the performance of the model to accurately classify breast cancer subtypes, we interpreted the attention scores of moBRCA-net. First, to directly compare the abundance difference between the feature subtypes with the highest attention scores, we visualized the normalized gene expression values and beta values of those features obtained from samples of each breast cancer subtype. One-way analysis of variance [45] was performed to test the statistical differences between the subtypes. From the results (Fig. 4, Additional file 1: S7), Overall, we could conclude that the features with the highest attention scores showed significant differences across the five subtypes with *p*-value $< 0.01$, indicating that the attention module trained moBRCA-net to assign more weights for the features having discriminative power for classifying the subtypes.

Studies have shown that DNA methylation regulates gene expression in breast cancer, with hypermethylation leading to the silencing of regulatory genes, whereas hypomethylation activates genes required for metastasis, consequently resulting in uncontrolled growth and cancer progression [46]. DNA methylation shows distinct patterns for each breast cancer subtype;thus, it has the potential to be used as a subtype-specific marker [47]. We hypothesized that the attention module would assign more weight to the biologically relevant features and identify the features showing a negative correlation ($<-0.5$) for each subtype. From each omics dataset, 200 features showing the highest average attention scores across patients were selected and the Pearson correlation between those features was analyzed. We identified feature pairs showing a negative correlation in different breast cancer subtypes, excluding the pairs of the normal-like subtype. NDRG2 showed a negative correlation with the CpG cluster composed of cg14030359 and cg18081258 in the basal subtype, and STAT5 showed negative correlation with the CpG cluster composed of cg03001305 and cg16777510 in the luminal A, B, and basal-like subtypes. These results were consistent with that of recent reports that showed that basal-like cancers more frequently present abundant NDRG2 expression in association with CpG-hypomethylation, with is associated with aggressiveness and unfavorable outcomes in the basal-like subtype [48]. STAT5 expression is a predictive factor for hormone therapy response and strong prognostic molecular marker in ER-positive breast cancer [49]. Indeed, STAT5 was shown to be significantly downregulated in patients with basal-like cancers [50]. Furthermore, it has been reported that the DNA methylation pattern of cg03001305 is associated with STAT5 in low-density cholesterol [51]. Similar investigations were performed regarding the herein selected microRNAs to identify negative correlations between them and target genes; however, significant results were difficult to find, which could be due to the relatively small number of microRNAs included in our framework compared with other omics studies. Top 200 features showing the highest average attention scores across patients for each omics were provided in Additional file 1: S8.

In addition, we used the Molecular Signature Database (MSigDB) [52, 53] collections and PAM50 [6] gene sets to explore whether the top 200 genes showing the highest average attention scores across patients have overlap with those known gene sets. From MSigDB, 10 types of gene sets [54] were obtained, where each set consists of genes either up or down-regulated in each breast cancer subtype. For PAM50 genes, during the multi-omics data integration step, most of the genes were filtered out, and only 18 genes were left as the input for the classifier. As a result, 53 genes of the top 200 genes were the functional genes known for the breast cancer subtype (51 genes from MSigDB and 2 genes from PAM 50). We listed the overlapped genes with the corresponding gene set collection name in the Additional file 1: S9.

## Discussion and conclusion

In this study, we describe moBRCA-net, a self-attention-based breast cancer subtype classification framework based on multi-omics integration. Gene expression, DNA methylation, and microRNA expression were integrated considering their biological relationships, and an omics-level attention module was implemented to identify and learn the important features critical for cancer classification. The model was then evaluated and compared with ML-based established methods. Overall, moBRCA-net outperformed the previously reported models in breast cancer subtype prediction. Moreover, we investigated the effectiveness of each module in moBRCA-net for classification improvement. Attention scores were interpreted to validate whether our model could concentrate on features highly relevant to cancer subtype classification.

The major contribution of our study resides within the multi-omics data integration strategy. To maintain the biological relationship between the multi omics features while integration, feature selection module was constructed to identify the informative breast cancer signature genes and the relation between the identified genes and other two omics features were built based on the promoter and the target relationship. Moreover, we employed the omics-level attention module to first train the classifier to capture the relative importance of each feature for each omics data. Features were transformed to new representations considering the respective learned importance. These lead the breast cancer subtype classifier not to ignore the distribution difference of each omics dataset and to integrate multi-omics more efficiently by reducing the dimensionality of each omics. This is also supported by the experiments performed in our study (Fig. 2 in the “Evaluation of moBRCA-net performance” Section) as well as the experiments shown in the MOGONET and MOMA paper presented for disease classification tasks, where the variant of the proposed classifiers without the omics-specific learning showed less prediction performance compared to the original models.

moBRCA-net showed a high prediction performance, but there are still some bottlenecks that could be further improved. For example, due to the limitation of the computational resources, feature selection was performed to reduce the number of features for training our model. Also, if the model could learn the dependency between the omics features directly via graph network, it could potentially be able to extract useful relations between the features of different omics datasets. In the future, we plan to extend our moBRCA-net platform to utilize graph neural networks.

## Supplementary Information

**Additional file 1: S1: Table 1.** Hyperparmeter optimization results based on the average accuracy for classifying breast cancer subtypes.** Table 2.** Classification accuracy results with the different number of selected features in multi-omics data integration.** S2:** Optimization results of the baseline methods with the different combination of the parameters.** S3:** Accuracy and weighted F1-score results of moBRCA-net, its variants, and the machine learning-based classifiers from the performance evaluation based on 10-fold cross validation in Fig. 2. **S4:** Average subtype-wise accuracy and weighted F1-score results of moBRCA-net (omics-attn) from the performance evaluation based on 10-fold cross validation in Fig. 2. **S5:** Average overall and subtype-wise performance results of moBRCA-net with/without data augmentation based on 10-fold cross validation.** S6:** Accuracy and weighted F1-score results of moBRCA-net using different feature selection methods in the multiomics data integration based on 10-fold cross validation in Fig. 3.** S7:** Normalized values of features showing top 3 to 5 average attention scores from each omics.** S8:** Top 200 features showing the highest average attention scores across patients for each omics dataset.** S9:** 53 overlapped genes with the gene set collection from MSigDB and PAM50, amont the top 200 genes showing the highest avearge attention scores across patients.

## Data Availability

TCGA datasets are publicly available from GDC Data Portal (https://portal.gdc.cancer.gov/). In this paper, ’TCGA-BRCA’ project datasets (dbGaP Study Accession: phs000178) were used, where three types of omics datasets (Gene expression, DNA methylation, and microRNA expression) were utilized. moBRCA-net is publicly available at https://github.com/cbi-bioinfo/moBRCA-net.

## Abbreviations

CNV: Copy number variation
BRCA: Breast cancer
TCGA: The Cancer Genome Atlas
DEG: Differentially expressed gene
Adam: Adaptive moment estimation
SVM: Support vector machine
RF: Random forest
LR: Logistic regression
NB: Naive Bayes
ML: Machine learning
SVM-RFE: Support vector machine-recursive feature elimination
