# Supplementary material for: moBRCA-net: a breast cancer subtype classification framework based on multi-omics attention neural networks
Source: BMC Bioinformatics. 2023 Apr 26;24:169. doi: 10.1186/s12859-023-05273-5 (PMC10131354; doi:10.1186/s12859-023-05273-5)
Supplement: Supplementary file 1 — Additional file 1: S1: Table 1. Hyperparmeter optimization results based on the average accuracy for classifying breast cancer subtypes. Table 2. Classification accuracy results with the different number of selected features in multi-omics data integration. S2: Optimization results of the baseline methods with the different combination of the parameters. S3: Accuracy and weighted F1-score results of moBRCA-net, its variants, and the machine learning-based classifiers from the performance evaluation based on 10-fold cross validation in Fig. 2. S4: Average subtype-wise accuracy and weighted F1-score results of moBRCA-net (omics-attn) from the performance evaluation based on 10-fold cross validation in Fig. 2. S5: Average overall and subtype-wise performance results of moBRCA-net with/without data augmentation based on 10-fold cross validation. S6: Accuracy and weighted F1-score results of moBRCA-net using different feature selection methods in the multiomics data integration based on 10-fold cross validation in Fig. 3. S7: Normalized values of features showing top 3 to 5 average attention scores from each omics. S8: Top 200 features showing the highest average attention scores across patients for each omics dataset. S9: 53 overlapped genes with the gene set collection from MSigDB and PAM50, amont the top 200 genes showing the highest avearge attention scores across patients. [file 12859_2023_5273_MOESM1_ESM.pdf]

SupplementaryMaterial S1.

Table1. Hyperparmeter optimization results based on the average accuracy for classifying breast cancer subtypes.

Learning rate	Dimension of embedding vector	Dimension of encodig vector	# of hidden nodes in classification module	Dropout	Average accuracy
1e-3	64	32	50	0.7	0.8679
1e-3	64	32	100	0.6	0.8490
1e-3	64	32	100	0.7	0.8773
1e-2	64	32	100	0.7	0.8679
1e-2	64	32	200	0.8	0.8490
1e-3	64	32	300	0.6	0.8584
1e-2	128	32	200	0.7	0.8490
1e-2	128	64	50	0.7	0.8773
1e-3	128	64	100	0.7	0.8773
1e-2	128	64	100	0.7	0.9230
1e-3	128	64	200	0.6	0.8679
1e-3	128	64	200	0.7	0.9079
**1e-2**	**128**	**64**	**200**	**0.7**	**0.9433**
1e-2	128	64	200	0.8	0.8867
1e-2	128	64	300	0.7	0.8974
1e-3	256	128	100	0.7	0.8679
1e-3	256	128	200	0.7	0.8679

Table2. Classification accuracy results with the different number of selected features in multi-omics data integration.

Number of selected DEGs	Number of features in the integrated multi-omics dataset			Average accuracy
	Gene expression	DNA methylation	microRNA	
800	683	683	213	0.9142
1000	835	835	228	0.9433
1500	1078	1078	229	0.8962

Supplementary Material S2.
Optimization results of the baseline methods with the different combination of the parameters. Grid search was adopted for the model tuning, and the hyperparameters showing the best accuracy were selected. Row with the bolded font are the hyperparmeters selected.

Support vector machine (SVM)

Kernel	Penalty parameter (C)	RBF kernel coeff (Gamma)	Accruacy
RBF	2^{-5}	2^{-15}	0.478
RBF	2^{-5}	2^{-13}	0.478
RBF	2^{-5}	2^{-11}	0.478
RBF	2^{-5}	2^{-9}	0.478
RBF	2^{-5}	2^{-7}	0.478
RBF	2^{-5}	2^{-5}	0.478
RBF	2^{-5}	2^{-3}	0.478
RBF	2^{-5}	2^{-1}	0.478
RBF	2^{-5}	2^{1}	0.478
RBF	2^{-5}	2^{3}	0.478
RBF	2^{-3}	2^{-15}	0.478
RBF	2^{-3}	2^{-13}	0.478
RBF	2^{-3}	2^{-11}	0.478
RBF	2^{-3}	2^{-9}	0.478
RBF	2^{-3}	2^{-7}	0.6384
RBF	2^{-3}	2^{-5}	0.6635
RBF	2^{-3}	2^{-3}	0.478
RBF	2^{-3}	2^{-1}	0.478
RBF	2^{-3}	2^{1}	0.478
RBF	2^{-3}	2^{3}	0.478
RBF	2^{-1}	2^{-15}	0.478
RBF	2^{-1}	2^{-13}	0.478
RBF	2^{-1}	2^{-11}	0.478
RBF	2^{-1}	2^{-9}	0.6447
RBF	2^{-1}	2^{-7}	0.739
RBF	2^{-1}	2^{-5}	0.783
RBF	2^{-1}	2^{-3}	0.5975
RBF	2^{-1}	2^{-1}	0.478
RBF	2^{-1}	2^{1}	0.478
RBF	2^{-1}	2^{3}	0.478
RBF	2^{1}	2^{-15}	0.478
RBF	2^{1}	2^{-13}	0.478
RBF	2^{1}	2^{-11}	0.6447
RBF	2^{1}	2^{-9}	0.7516
RBF	2^{1}	2^{-7}	0.8082
RBF	2^{1}	2^{-5}	0.8239
RBF	2^{1}	2^{-3}	0.7107
RBF	2^{1}	2^{-1}	0.478
RBF	2^{1}	2^{1}	0.478
RBF	2^{1}	2^{3}	0.478
RBF	2^{3}	2^{-15}	0.478
RBF	2^{3}	2^{-13}	0.6447
RBF	2^{3}	2^{-11}	0.7547

RBF	2^{3}	2^{-9}	0.8113
**RBF**	$\mathbf{2^{3}}$	$\mathbf{2^{-7}}$	**0.8365**
RBF	2^{3}	2^{-5}	0.827
RBF	2^{3}	2^{-3}	0.7107
RBF	2^{3}	2^{-1}	0.478
RBF	2^{3}	2^{1}	0.478
RBF	2^{3}	2^{3}	0.478
RBF	2^{5}	2^{-15}	0.6447
RBF	2^{5}	2^{-13}	0.7547
RBF	2^{5}	2^{-11}	0.8113
RBF	2^{5}	2^{-9}	0.8333
RBF	2^{5}	2^{-7}	0.8365
RBF	2^{5}	2^{-5}	0.827
RBF	2^{5}	2^{-3}	0.7107
RBF	2^{5}	2^{-1}	0.478
RBF	2^{5}	2^{1}	0.478
RBF	2^{5}	2^{3}	0.478
Linear	2^{-5}	-	0.478
Linear	2^{-3}	-	0.478
Linear	2^{-1}	-	0.478
Linear	2^{1}	-	0.6447
Linear	2^{3}	-	0.478
Linear	2^{5}	-	0.4780

Random Forest (RF)

Split criteria (criterion)	# of trees (estimators)	The minimum # of samples in a leaf node (min_samples_leaf)	Accruacy
Gini impurity	100	1	0.7925
Gini impurity	100	2	0.7830
**Gini impurity**	**100**	**3**	**0.8082**
Gini impurity	100	4	0.7799
Gini impurity	100	5	0.7925
Gini impurity	300	1	0.7987
Gini impurity	300	2	0.7925
Gini impurity	300	3	0.7925
Gini impurity	300	4	0.7925
Gini impurity	300	5	0.7799
Gini impurity	500	1	0.8082
Gini impurity	500	2	0.7956
Gini impurity	500	3	0.7987
Gini impurity	500	4	0.7893
Gini impurity	500	5	0.7956
Gini impurity	700	1	0.8019
Gini impurity	700	2	0.7956
Gini impurity	700	3	0.7956
Gini impurity	700	4	0.7925
Gini impurity	700	5	0.7862
Gini impurity	900	1	0.7925
Gini impurity	900	2	0.7956

Gini impurity	900	3	0.7925
Gini impurity	900	4	0.7862
Gini impurity	900	5	0.7799

Logistic Regression (LR)

max_iter (maximum number of iterations to converge)	Penalty parameter (C)	Accuracy
100	0.03125	0.7799
100	0.125	0.8239
100	0.5	0.827
100	1	0.8239
**100**	**2**	**0.8333**
100	8	0.8302
100	32	0.827
200	0.03125	0.7799
200	0.125	0.8239
200	0.5	0.827
200	1	0.8239
200	2	0.8333
200	8	0.8302
200	32	0.827
300	0.03125	0.7799
300	0.125	0.8239
300	0.5	0.827
300	1	0.8239
300	2	0.8333
300	8	0.8302
300	32	0.827
400	0.03125	0.7799
400	0.125	0.8239
400	0.5	0.827
400	1	0.8239
400	2	0.8333
400	8	0.8302
400	32	0.827
500	0.03125	0.7799
500	0.125	0.8239
500	0.5	0.827
500	1	0.8239
500	2	0.8333
500	8	0.8302
500	32	0.827

SupplementaryMaterial S3.

Accuracy and weighted F1-score results of moBRCA-net, its variants, and the machine learning-based classifiers from the performance evaluation based on 10-fold cross validation in Fig 2.

[Accuracy]

	moBRCA-net (omics-attn)	moBRCA-net (single attn)	moBRCA-net (no attn)	SVM	RF	LR	NB
v1	0.8868	0.8962	0.8962	0.8868	0.8019	0.8774	0.7547
v2	0.8585	0.8396	0.8302	0.8302	0.8113	0.8113	0.7547
v3	0.8585	0.8679	0.8396	0.8208	0.7925	0.8113	0.7264
v4	0.934	0.9245	0.8962	0.9057	0.8585	0.8774	0.6981
v5	0.9057	0.8396	0.8302	0.8868	0.8302	0.8113	0.7547
v6	0.8962	0.9057	0.9057	0.8585	0.8585	0.8774	0.717
v7	0.9057	0.8396	0.8962	0.8679	0.8585	0.8396	0.6792
v8	0.8868	0.9151	0.9151	0.8679	0.7925	0.8868	0.7358
v9	0.9151	0.8962	0.8302	0.9057	0.8585	0.8774	0.7547
v10	0.8667	0.8476	0.8476	0.8286	0.819	0.819	0.6857
**Average**	0.8914	0.8772	0.8687	0.8659	0.8281	0.8489	0.7261

[Weighted F1-score]

	moBRCA-net (omics-attn)	moBRCA-net (single attn)	moBRCA-net (no attn)	SVM	RF	LR	NB
v1	0.8842	0.8952	0.8952	0.8826	0.7673	0.8738	0.7457
v2	0.8556	0.8321	0.8263	0.8274	0.7925	0.8046	0.7728
v3	0.842	0.8602	0.8292	0.8096	0.759	0.7989	0.7405
v4	0.9338	0.9119	0.8893	0.9017	0.8397	0.8251	0.7078
v5	0.9002	0.8321	0.8263	0.8908	0.8186	0.8643	0.7644
v6	0.8899	0.9021	0.9051	0.8449	0.8278	0.8643	0.7321
v7	0.9052	0.8321	0.8893	0.8605	0.8381	0.8251	0.6895
v8	0.8778	0.9123	0.9123	0.8626	0.7461	0.8624	0.7471
v9	0.9158	0.8936	0.8263	0.9023	0.8421	0.875	0.773
v10	0.8612	0.8518	0.8364	0.83	0.8157	0.813	0.6975
**Average**	0.8866	0.8723	0.8636	0.8612	0.8047	0.8407	0.7370

[MCC]

	moBRCA-net (omics-attn)	moBRCA-net (single attn)	moBRCA-net (no attn)	SVM	RF	LR	NB
v1	0.8413	0.8527	0.8527	0.7826	0.6844	0.7297	0.667
v2	0.7854	0.755	0.7387	0.8473	0.7829	0.7831	0.6316
v3	0.7827	0.8005	0.7579	0.7985	0.7676	0.7987	0.6183
v4	0.8939	0.877	0.8601	0.822	0.7743	0.7608	0.5536
v5	0.8242	0.8242	0.867	0.8462	0.8153	0.8407	0.6562
v6	0.8401	0.8541	0.8564	0.8106	0.7162	0.7794	0.6201
v7	0.8625	0.8625	0.8472	0.8206	0.7733	0.7944	0.5302
v8	0.8327	0.8737	0.8737	0.821	0.8675	0.8349	0.5809
v9	0.8671	0.836	0.836	0.7241	0.6948	0.7373	0.6453
v10	0.7759	0.7573	0.7426	0.8106	0.7707	0.7552	0.5997
**Average**	0.8306	0.8293	0.8232	0.8084	0.7647	0.7814	0.6103

SupplementaryMaterial S4.

Average subtype-wise accuracy and weighted F1-score results of moBRCA-net (omics-attn) from the performance evaluation based on 10-fold cross validation in Fig 2.

Subtype	Accuracy	F1-score
Basal	0.9244	0.9289
Her2	0.9292	0.7942
Luminal A	0.9603	0.7254
Luminal B	0.9896	0.9737
Normal-like	0.5967	0.6099

SupplementaryMaterial S5.

Average overall and subtype-wise performance results of moBRCA-net with/without data augmentation based on 10-fold cross validation.

	Overall			Luminal A		Luminal B		Basal		Her2		Normal-like	
	ACC	F1	MCC	ACC	F1	ACC	F1	ACC	F1	ACC	F1	ACC	F1
w/o aug	0.891	0.887	0.831	0.96	0.725	0.989	0.974	0.924	0.928	0.929	0.794	0.596	0.609
with aug	0.895	0.891	0.84	0.966	0.763	0.987	0.966	0.927	0.929	0.93	0.819	0.599	0.618

SupplementaryMaterial S6.

Accuracy and weighted F1-score results of moBRCA-net using different feature selection methods in the multi-omics data integration based on 10-fold cross validation in Fig 3.

[Accuracy]

	**CpG cluster**			**Single CpG**		
	DEG	SVM-RFE	RF	DEG	SVM-RFE	RF
v1	0.8962	0.8491	0.8396	0.8585	0.8396	0.8208
v2	0.8962	0.8208	0.8396	0.8491	0.8208	0.8396
v3	0.8679	0.8585	0.8491	0.8585	0.8396	0.8302
v4	0.9434	0.9151	0.9057	0.8962	0.8962	0.8208
v5	0.9245	0.8774	0.8868	0.8585	0.8962	0.8585
v6	0.9434	0.9057	0.8868	0.9057	0.8868	0.8774
v7	0.9057	0.8774	0.8774	0.8679	0.8962	0.8774
v8	0.9151	0.8774	0.8396	0.8491	0.8868	0.8396
v9	0.9151	0.8679	0.8868	0.8868	0.8302	0.8585
v10	0.8857	0.8571	0.8952	0.8381	0.8667	0.8774
**Average**	0.9093	0.8706	0.8707	0.8668	0.8659	0.8500

[Weighted F1-score]

	**CpG cluster**			**Single CpG**		
	DEG	SVM-RFE	RF	DEG	SVM-RFE	RF
v1	0.8952	0.8464	0.8307	0.8518	0.8307	0.8227
v2	0.8937	0.8230	0.8320	0.8431	0.8164	0.8320
v3	0.8602	0.8502	0.8464	0.8450	0.8332	0.8222
v4	0.9426	0.9108	0.8985	0.8885	0.8901	0.8227
v5	0.9268	0.8792	0.8861	0.8585	0.8884	0.8530
v6	0.9448	0.9004	0.8861	0.9121	0.8861	0.8700
v7	0.9052	0.8728	0.8698	0.8672	0.8933	0.8698
v8	0.9123	0.8786	0.8319	0.8415	0.8832	0.8319
v9	0.9158	0.8625	0.8832	0.8764	0.8135	0.8537
v10	0.8829	0.8341	0.8951	0.8515	0.8651	0.8698
**Average**	0.9080	0.8658	0.8660	0.8636	0.8600	0.8448

SupplementaryMaterial S7. Normalized values of features showing top 3 to 5 average attention scores from each omics

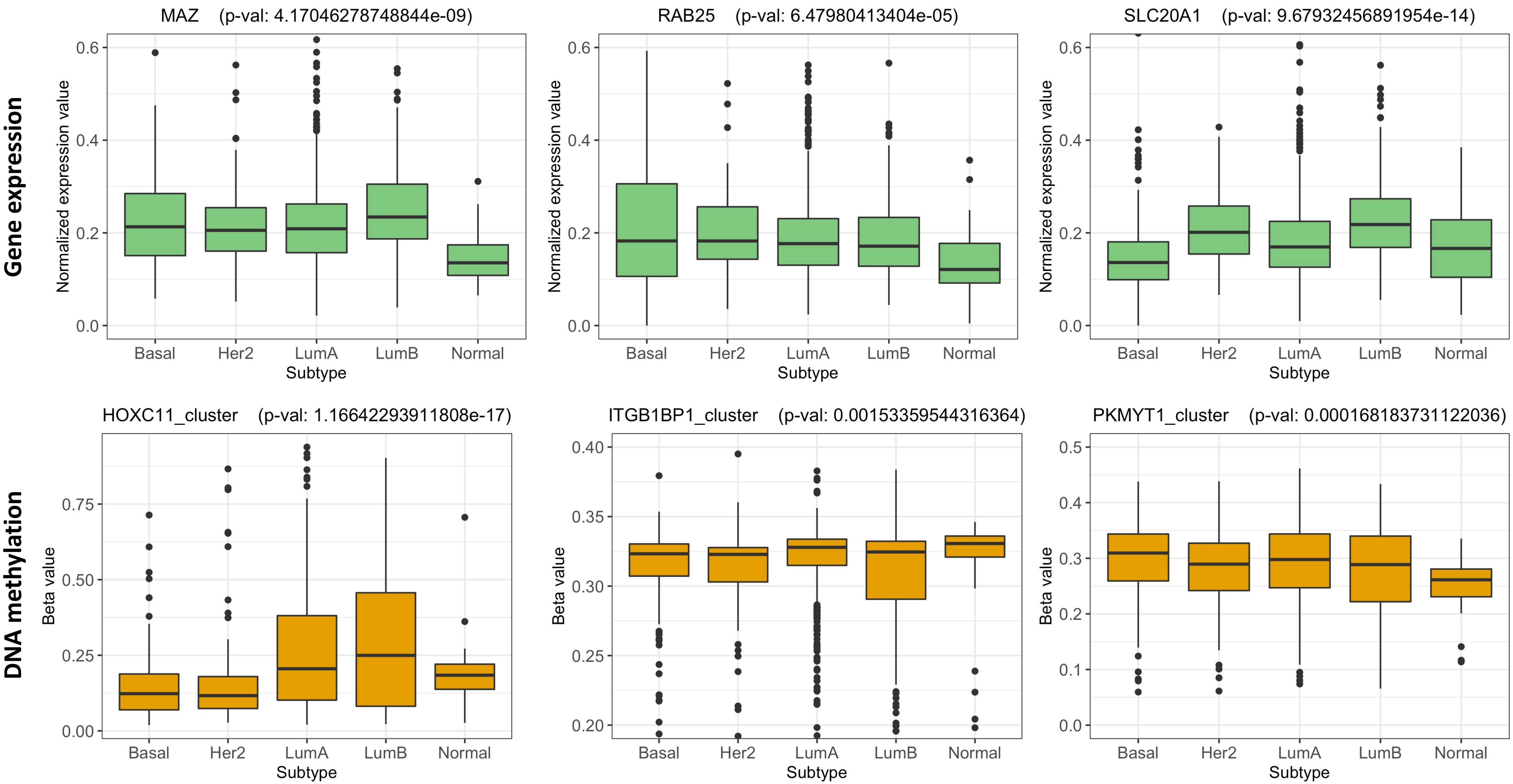

SupplementaryMaterial S7. Normalized values of features showing top 3 to 5 average attention scores from each omics

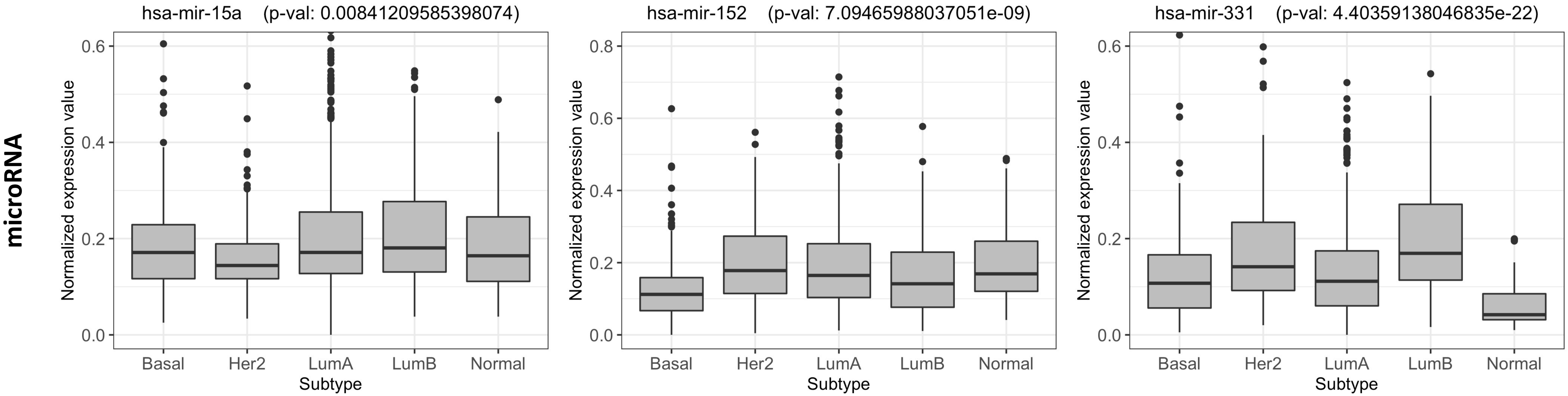

SupplementaryMaterial S8.

Top 200 features showing the highest average attention scores across patients for each omics dataset.

Gene
EZH1
HDGF
MAZ
RAB25
SLC20A1
TK2
STAT5B
TJP3
ESRP1
SLC25A39
TIMM17A
AHNAK
GPRASP2
EHD2
NKAIN1
TPM3
NACC1
PDP2
LIG1
CBX4
RETSAT
STMN1
JTB
RBP7
PCYOX1
ZNF48
SEPHS2
KIF15
RHOQ
ASPM
ZCCHC24
FERMT2
TROAP
CDCA4
KIF22
ESPL1
HNRNPAB
TARS2
HIST1H2BD
CHEK1
LTBP4
SNTB2
KLF9
TMEM140
TUBA1C
MRAS
TOR3A

KANK1
NEK2
ATAD2
ERG
MMRN2
TMEM8A
SNRNP25
EHBP1
FAM126A
MKI67
PPP1CA
CENPI
GSN
PPP4C
KIF24
ARHGEF15
KCTD13
ACAA2
CLEC5A
MGLL
WDR34
TRIB3
PER2
MCM2
ITGB1BP1
GTF2IRD1
CKAP2L
SORBS1
KIF23
ZWINT
H2AFZ
NDC80
CDK5
KAT2B
POLR2H
MICAL2
SDC1
ADAM8
STBD1
CCT3
NR3C1
CANT1
HOXC13
NUP210
H2AFY
TEF
SRMS
GPRASP1
CSRP1
CACNB3
CEP68

CENPO
PACSIN1
EPM2A
RUVBL1
F10
NR2F6
MANF
SPC25
IQGAP3
SCAMP3
STAT5A
RAD51
RABIF
LMNB1
KIF4A
DLC1
MAML2
SPINT2
RAD54L
ELMOD3
HELLS
FLAD1
LRWD1
MFAP4
GPR68
ROGDI
DLGAP5
CCDC69
PNPLA2
ST6GALNAC6
LMOD1
TLN2
GYG2
FOXP3
RELT
B4GALT3
NDRG2
ARF1
STRBP
CENPE
INHBA
PKD2
PPP1R12B
SH3BGRL2
SLC25A22
XRCC2
MMD
LEPR
RUSC1
AMOTL2
TGFBR3

KLF11
BCL9
C2CD2
LDB2
LRRN4CL
ANO6
TRAIP
HABP4
RNASEH2A
CFL1
GPRIN1
REEP4
ADHFE1
RCC2
FKBP4
THSD1
VAV2
TSTA3
RNFT2
FADS3
NAT8L
JAM2
GGCT
KLF6
TXNIP
RBMS2
PLXNA3
SPTBN1
C9orf116
KL
TNFSF4
LEF1
DNASE1L2
CALCOCO1
FOXN3
FBXL6
CTHRC1
TMEM206
CDCA8
RCBTB2
STX11
PDLIM3
CDC25C
MAPK8IP2
EPB41L2
CAT
HM13
GTSE1
DAB2IP
TMED3
KLF10

ClusterName	cpg
TNS1_cluster	cg18328334
SLC22A3_cluster	cg07237939
SLC22A3_cluster	cg25313204
HOXC11_cluster	cg07123069
HOXC11_cluster	cg22709192
ITGB1BP1_cluster	cg00024812
ITGB1BP1_cluster	cg23889771
ITGB1BP1_cluster	cg26306976
PKMYT1_cluster	cg00319761
PKMYT1_cluster	cg02510853
ADIPOQ_cluster	cg03573747
CA9_cluster	cg19257550
CA9_cluster	cg20610181
MCAM_cluster	cg03365354
MCAM_cluster	cg21096399
NRN1_cluster	cg11504897
NRN1_cluster	cg25511429
RASSF7_cluster	cg00216758
RASSF7_cluster	cg14896003
RASSF7_cluster	cg16000888
GABRD_cluster	cg06063714
GABRD_cluster	cg24087944
HOXC13_cluster	cg20587394
KCTD12_cluster	cg12280407
KCTD12_cluster	cg15901783
KRT8_cluster	cg01835489
KRT8_cluster	cg20324165
CBX2_cluster	cg06162003
CBX2_cluster	cg22892904
TPD52_cluster	cg18459342
TMC2_cluster	cg03243506
TMC2_cluster	cg19290962
GNG11_cluster	cg22983529
FAM110A_cluster	cg20289911
DEFB132_cluster	cg18239253
ERG_cluster	cg03127334
ERG_cluster	cg17274064
MYBL2_cluster	cg09604203
MYBL2_cluster	cg23843505
ADAMTS5_cluster	cg08190291
ADAMTS5_cluster	cg18895813
ESPL1_cluster	cg03815350
ESPL1_cluster	cg13445358
CDH13_cluster	cg00806490
CDH13_cluster	cg01880569
CDH13_cluster	cg08747377
CDH13_cluster	cg08977371
CDH13_cluster	cg19369556

CDH13_cluster	cg19854301
MMP11_cluster	cg27532722
SAA1_cluster	cg15484375
FGF2_cluster	cg17214107
FGF2_cluster	cg25583174
HMGB3_cluster	cg05935584
MASP1_cluster	cg20725021
MASP1_cluster	cg21831174
MS4A15_cluster	cg19105504
MS4A15_cluster	cg25072962
MUC1_cluster	cg07399355
MUC1_cluster	cg17257175
MUC1_cluster	cg24512973
FMO2_cluster	cg14721213
MME_cluster	cg16580737
MME_cluster	cg23273897
CCT3_cluster	cg18388972
CCT3_cluster	cg18993334
CCT3_cluster	cg24042452
GSN_cluster	cg17071957
RECQL4_cluster	cg03144357
RECQL4_cluster	cg15857475
RECQL4_cluster	cg21092687
TRDN_cluster	cg14462830
SYNGR3_cluster	cg26486663
PLSCR4_cluster	cg04959837
PLSCR4_cluster	cg24315815
VGF_cluster	cg04084157
VGF_cluster	cg08097755
CCDC69_cluster	cg08317263
PACSIN1_cluster	cg23043245
PACSIN1_cluster	cg23239444
TGFBR3_cluster	cg00563926
TGFBR3_cluster	cg16382382
COL1A1_cluster	cg01234133
COL1A1_cluster	cg01593886
BUB3_cluster	cg04058169
BUB3_cluster	cg23724447
SLC7A10_cluster	cg00910067
SLC7A10_cluster	cg05976074
CHRNA6_cluster	cg07906724
TSTA3_cluster	cg00311768
TSTA3_cluster	cg19150884
CIB3_cluster	cg09424896
CIB3_cluster	cg09686308
FAM49A_cluster	cg10106284
FAM49A_cluster	cg24242519
DST_cluster	cg04452713
CILP2_cluster	cg10313673
CILP2_cluster	cg10669058
EPAS1_cluster	cg17518825

TJP3_cluster	cg27022827
PRAME_cluster	cg05208878
PAX7_cluster	cg07536847
PAX7_cluster	cg11428724
NAT8L_cluster	cg21269934
TMEM8A_cluster	cg01940867
TMEM8A_cluster	cg10805676
TSLP_cluster	cg15089387
TSLP_cluster	cg15739437
ESM1_cluster	cg07233761
ESM1_cluster	cg20451680
AOC3_cluster	cg09040752
AOC3_cluster	cg21602160
COMP_cluster	cg09949775
COMP_cluster	cg15784332
EGR2_cluster	cg19355190
CA3_cluster	cg18674980
CA3_cluster	cg22289837
CHL1_cluster	cg00903242
NKX3-2_cluster	cg11392765
NKX3-2_cluster	cg20073553
ENPP2_cluster	cg14409958
PPP1CA_cluster	cg13055001
ATP1A3_cluster	cg07051960
ATP1A3_cluster	cg23920441
ANK2_cluster	cg02735486
TAL1_cluster	cg00875272
TAL1_cluster	cg19797376
HIF3A_cluster	cg02879662
PRKAR2B_cluster	cg21250978
STAT5A_cluster	cg03001305
STAT5A_cluster	cg16777510
MRGPRF_cluster	cg22933847
MRGPRF_cluster	cg22986999
CA4_cluster	cg04532952
CA4_cluster	cg18743730
CSAG1_cluster	cg02504280
HOXB13_cluster	cg15786837
HOXB13_cluster	cg21842478
PDE3B_cluster	cg03439703
PDE3B_cluster	cg05532892
PDE3B_cluster	cg14400631
PDE3B_cluster	cg27143049
GNG13_cluster	cg14100184
BGN_cluster	cg19618706
PAK3_cluster	cg14688956
PAK3_cluster	cg14708847
CRIM1_cluster	cg00850538
CRIM1_cluster	cg10881306
APCDD1_cluster	cg19264571
APCDD1_cluster	cg26328002

FBXL6_cluster	cg16628918
FBXL6_cluster	cg21959619
FBXL6_cluster	cg26403198
PNPLA2_cluster	cg22016649
PNPLA2_cluster	cg24427660
LGALS12_cluster	cg11484576
HIST1H3J_cluster	cg17965019
CELSR3_cluster	cg06621358
CELSR3_cluster	cg09736162
MATN2_cluster	cg10039928
MATN2_cluster	cg16202564
SRPX_cluster	cg23750556
LILRB5_cluster	cg08684473
LILRB5_cluster	cg20649991
CCR8_cluster	cg26647600
HAGHL_cluster	cg10163825
RECK_cluster	cg03081134
RECK_cluster	cg12717594
ADCY4_cluster	cg12265829
ADCY4_cluster	cg16761581
ADAM33_cluster	cg14089692
ADAM33_cluster	cg22333888
RNASEH2A_cluster	cg05657090
RNASEH2A_cluster	cg24056562
EDNRB_cluster	cg03086857
EDNRB_cluster	cg12120741
EDNRB_cluster	cg12847373
EDNRB_cluster	cg15836660
EDNRB_cluster	cg23316360
EDNRB_cluster	cg24745738
SLC29A4_cluster	cg05168404
CCDC3_cluster	cg22750254
CLEC1A_cluster	cg07959477
CLEC1A_cluster	cg13354523
CHRNA9_cluster	cg10375110
CEP55_cluster	cg24371383
ALDH1L1_cluster	cg07800320
ALDH1L1_cluster	cg16312163
SCN4B_cluster	cg17186803
SCN4B_cluster	cg27560864
DMRT2_cluster	cg00250430
DMRT2_cluster	cg11530960
ATP6AP1_cluster	cg18742441
PKD2_cluster	cg23916751
PKD2_cluster	cg26215428
BMP8A_cluster	cg03664992
PPP4C_cluster	cg11970301
PPP4C_cluster	cg21742836
CASP14_cluster	cg01999333
HAPLN1_cluster	cg06434428
HAPLN1_cluster	cg09893305

LDB2_cluster	cg03368758
LDB2_cluster	cg08899626
HSD17B7_cluster	cg04469686
HSD17B7_cluster	cg07774025
SOX17_cluster	cg02919422
LEP_cluster	cg12782180
LEP_cluster	cg19594666
CEACAM6_cluster	cg26813458
CIDEA_cluster	cg19883905
CIDEA_cluster	cg20950011
MAOA_cluster	cg15014034
MAOA_cluster	cg19441691
ARHGEF15_cluster	cg12989650
ARHGEF15_cluster	cg19537511
KIF4A_cluster	cg16256230
GINS2_cluster	cg19890739
GINS2_cluster	cg23871933
RRM2_cluster	cg02237186
RRM2_cluster	cg18623836
AGPAT2_cluster	cg15720535
AGPAT2_cluster	cg25537774
SPARCL1_cluster	cg19466563
HSD17B13_cluster	cg08815403
HSD17B13_cluster	cg24999727
DDR2_cluster	cg22740835
IRF7_cluster	cg00645579
IRF7_cluster	cg16541031
HBA1_cluster	cg10647513
PTK6_cluster	cg21484834
CD80_cluster	cg21572897
CRB3_cluster	cg03258472
NNAT_cluster	cg23566503
TSPAN7_cluster	cg20450764
TSPAN7_cluster	cg21516819
HIST1H2BJ_cluster	cg04424621
HIST1H2BJ_cluster	cg08717396
HOXA5_cluster	cg02248486
HOXA5_cluster	cg12128839
SLC25A39_cluster	cg03192551
SLC25A39_cluster	cg24497270
CBX4_cluster	cg04398978
CBX4_cluster	cg17533847
NDRG2_cluster	cg14030359
NDRG2_cluster	cg18081258
MAB21L1_cluster	cg05093686
PDK4_cluster	cg04498739
PDK4_cluster	cg22171829
HIST1H2AI_cluster	cg10046620
TNNT1_cluster	cg07189381
TNNT1_cluster	cg19504245
RGL1_cluster	cg03125090

RGL1_cluster	cg11505048
TNXB_cluster	cg03543593
TNXB_cluster	cg13823701
ARTN_cluster	cg06207804
ARTN_cluster	cg22930187
MYT1_cluster	cg10071275
KLB_cluster	cg21880903
KLB_cluster	cg27558666
GJB2_cluster	cg02861781
GJB2_cluster	cg11054936
GYPC_cluster	cg17105014
KLF11_cluster	cg02983451
NPR2_cluster	cg12876594
PER1_cluster	cg16545079
RBP7_cluster	cg03406535
NCAPH_cluster	cg01906055
NCAPH_cluster	cg26625319
SRMS_cluster	cg03330516
POLR3K_cluster	cg11876012
POLR3K_cluster	cg14820573
TMEM145_cluster	cg02456292
ANGPT1_cluster	cg09396217
CASQ2_cluster	cg05190718
CASQ2_cluster	cg18942631
IGF1_cluster	cg01305421
ORAI2_cluster	cg02564523
TPM3_cluster	cg24490338
KIT_cluster	cg27154163
CCL14_cluster	cg09256683
NOTCH4_cluster	cg05973262
NOTCH4_cluster	cg14700707
PAMR1_cluster	cg14542839
PAMR1_cluster	cg14642338
MARCKSL1_cluster	cg06434451
MARCKSL1_cluster	cg09327628
HEPACAM_cluster	cg06142324
CAPSL_cluster	cg10994379
CAPSL_cluster	cg24202119
COL11A1_cluster	cg12884406
COL11A1_cluster	cg26256793
FAM89A_cluster	cg00679738
FAM89A_cluster	cg16516400
JAM2_cluster	cg03382304
PLXNA3_cluster	cg07428182
LTBP4_cluster	cg25723866
IGFBP6_cluster	cg01773854
IGFBP6_cluster	cg08629913
IGFBP6_cluster	cg23841186
EBF3_cluster	cg06139836
TACC1_cluster	cg04017769
TACC1_cluster	cg09990086

FBN2_cluster	cg25084878
FBN2_cluster	cg27223047
TLX1_cluster	cg11498156
TLX1_cluster	cg16715722
GNAL_cluster	cg09331011
GNAL_cluster	cg14371329
GIPC2_cluster	cg09107315
GIPC2_cluster	cg24496666
RUSC1_cluster	cg11503744
RUSC1_cluster	cg22234962
RUSC1_cluster	cg23060239
AKAP6_cluster	cg24812523
NAALAD2_cluster	cg05500015
NAALAD2_cluster	cg21500966
TIMP4_cluster	cg25982743
HIST1H2BE_cluster	cg21146268
MYOM2_cluster	cg21132577
LAGE3_cluster	cg20641280
LAGE3_cluster	cg25410279
CXCL12_cluster	cg00499822
CXCL12_cluster	cg18618334
HLF_cluster	cg04219321
FGF1_cluster	cg08816023
FGF1_cluster	cg19954000
MAMDC2_cluster	cg11656547
MAMDC2_cluster	cg13870494
HTR1D_cluster	cg08872493
HTR1D_cluster	cg20887241
FBLN5_cluster	cg14974772
FBLN5_cluster	cg24777065
RDH5_cluster	cg02192520
RDH5_cluster	cg17243643
CNN1_cluster	cg12854483
KLHL4_cluster	cg07897414

**microRNA**
hsa-mir-99b
hsa-let-7d
hsa-mir-331
hsa-mir-15a
hsa-mir-152
hsa-mir-324
hsa-mir-151a
hsa-mir-200c
hsa-mir-374a
hsa-mir-200a
hsa-mir-190b
hsa-mir-146b
hsa-mir-22
hsa-mir-27a

hsa-mir-190a
hsa-let-7i
hsa-mir-98
hsa-mir-421
hsa-mir-1306
hsa-mir-342
hsa-mir-200b
hsa-mir-30b
hsa-mir-32
hsa-mir-3064
hsa-mir-93
hsa-mir-1249
hsa-mir-141
hsa-mir-23b
hsa-mir-326
hsa-mir-100
hsa-mir-106b
hsa-mir-29c
hsa-mir-365a
hsa-mir-21
hsa-mir-96
hsa-mir-134
hsa-mir-186
hsa-mir-145
hsa-mir-328
hsa-mir-29a
hsa-mir-34a
hsa-mir-148b
hsa-mir-338
hsa-mir-497
hsa-mir-346
hsa-mir-1271
hsa-mir-142
hsa-mir-149
hsa-mir-877
hsa-mir-155
hsa-mir-26b
hsa-mir-874
hsa-mir-503
hsa-mir-30e
hsa-mir-182
hsa-mir-542
hsa-mir-199b
hsa-mir-744
hsa-mir-379
hsa-mir-28
hsa-mir-378a
hsa-mir-424
hsa-mir-210
hsa-mir-455
hsa-mir-193a

hsa-mir-125a
hsa-mir-551b
hsa-mir-499a
hsa-let-7e
hsa-mir-301a
hsa-let-7g
hsa-mir-192
hsa-mir-146a
hsa-mir-188
hsa-mir-139
hsa-mir-191
hsa-mir-195
hsa-mir-23a
hsa-mir-20a
hsa-mir-30a
hsa-mir-760
hsa-mir-143
hsa-mir-502
hsa-mir-107
hsa-mir-205
hsa-mir-221
hsa-mir-215
hsa-mir-381
hsa-mir-330
hsa-mir-411
hsa-mir-99a
hsa-mir-454
hsa-mir-33a
hsa-mir-876
hsa-mir-377
hsa-mir-365b
hsa-mir-320a
hsa-mir-1224
hsa-mir-296
hsa-mir-185
hsa-mir-429
hsa-mir-615
hsa-mir-374b
hsa-mir-130a
hsa-mir-193b
hsa-mir-425
hsa-mir-15b
hsa-let-7b
hsa-mir-216b
hsa-mir-202
hsa-mir-30d
hsa-mir-135b
hsa-mir-582
hsa-mir-382
hsa-mir-33b
hsa-mir-501

hsa-mir-212
hsa-mir-361
hsa-mir-409
hsa-mir-10a
hsa-mir-19a
hsa-mir-27b
hsa-mir-339
hsa-mir-106a
hsa-mir-590
hsa-mir-708
hsa-mir-452
hsa-mir-17
hsa-mir-136
hsa-mir-551a
hsa-mir-1197
hsa-mir-491
hsa-mir-668
hsa-mir-148a
hsa-mir-129-1
hsa-mir-449b
hsa-mir-92b
hsa-mir-448
hsa-mir-362
hsa-mir-222
hsa-mir-214
hsa-mir-363
hsa-mir-490
hsa-mir-140
hsa-mir-340
hsa-mir-25
hsa-mir-1251
hsa-mir-335
hsa-mir-130b
hsa-mir-223
hsa-mir-410
hsa-mir-494
hsa-mir-10b
hsa-mir-20b
hsa-mir-373
hsa-mir-758
hsa-let-7c
hsa-mir-655
hsa-mir-208a
hsa-mir-18a
hsa-mir-150
hsa-mir-506
hsa-mir-423
hsa-mir-6835
hsa-mir-532
hsa-mir-665
hsa-mir-18b

hsa-mir-1193
hsa-mir-154
hsa-mir-539
hsa-mir-224
hsa-mir-433
hsa-mir-301b
hsa-mir-375
hsa-mir-302a
hsa-mir-299
hsa-mir-181d
hsa-mir-196b
hsa-mir-34c
hsa-mir-369
hsa-mir-495
hsa-mir-493
hsa-mir-211
hsa-mir-219a-2
hsa-mir-543
hsa-mir-181c
hsa-mir-372
hsa-mir-184
hsa-mir-485
hsa-mir-129-2
hsa-mir-302b
hsa-mir-31
hsa-mir-323a
hsa-mir-431
hsa-mir-208b
hsa-mir-132
hsa-mir-23c
hsa-mir-488
hsa-mir-487a
hsa-mir-376b

SupplementaryMaterial S9.

53 overlapped genes with the gene set collection from MSigDB and PAM50, amont the top 200 genes showing the highest avearge attention scores across patients

Gene	Gene set collection name
TK2	SMID_BREAST_CANCER_BASAL_DN
TJP3	SMID_BREAST_CANCER_BASAL_DN
AHNAK	SMID_BREAST_CANCER_BASAL_DN
NKAIN1	SMID_BREAST_CANCER_BASAL_DN
ZCCHC24	SMID_BREAST_CANCER_BASAL_DN
CANT1	SMID_BREAST_CANCER_BASAL_DN
MFAP4	SMID_BREAST_CANCER_BASAL_DN
PNPLA2	SMID_BREAST_CANCER_BASAL_DN
ASPM	SMID_BREAST_CANCER_BASAL_UP
CHEK1	SMID_BREAST_CANCER_BASAL_UP
MRAS	SMID_BREAST_CANCER_BASAL_UP
NEK2	SMID_BREAST_CANCER_BASAL_UP
ATAD2	SMID_BREAST_CANCER_BASAL_UP
MKI67	SMID_BREAST_CANCER_BASAL_UP
MCM2	SMID_BREAST_CANCER_BASAL_UP
NDC80	SMID_BREAST_CANCER_BASAL_UP
RAD54L	SMID_BREAST_CANCER_BASAL_UP
HELLS	SMID_BREAST_CANCER_BASAL_UP
DLGAP5	SMID_BREAST_CANCER_BASAL_UP
NDRG2	SMID_BREAST_CANCER_BASAL_UP
CENPE	SMID_BREAST_CANCER_BASAL_UP
KLF6	SMID_BREAST_CANCER_BASAL_UP
CDCA8	SMID_BREAST_CANCER_BASAL_UP
EPB41L2	SMID_BREAST_CANCER_BASAL_UP
GTSE1	SMID_BREAST_CANCER_BASAL_UP
NDC80	SMID_BREAST_CANCER_LUMINAL_A_DN
CDCA8	SMID_BREAST_CANCER_LUMINAL_A_DN
MFAP4	SMID_BREAST_CANCER_LUMINAL_A_UP
LMOD1	SMID_BREAST_CANCER_LUMINAL_A_UP
LEPR	SMID_BREAST_CANCER_LUMINAL_A_UP
FERMT2	SMID_BREAST_CANCER_LUMINAL_B_DN
MRAS	SMID_BREAST_CANCER_LUMINAL_B_DN
MFAP4	SMID_BREAST_CANCER_LUMINAL_B_DN
NDRG2	SMID_BREAST_CANCER_LUMINAL_B_DN
LEPR	SMID_BREAST_CANCER_LUMINAL_B_DN
KLF6	SMID_BREAST_CANCER_LUMINAL_B_DN
EPB41L2	SMID_BREAST_CANCER_LUMINAL_B_DN
TJP3	SMID_BREAST_CANCER_LUMINAL_B_UP
NKAIN1	SMID_BREAST_CANCER_LUMINAL_B_UP
EZH1	SMID_BREAST_CANCER_NORMAL_LIKE_UP
SORBS1	SMID_BREAST_CANCER_NORMAL_LIKE_UP
F10	SMID_BREAST_CANCER_NORMAL_LIKE_UP
MFAP4	SMID_BREAST_CANCER_NORMAL_LIKE_UP
CCDC69	SMID_BREAST_CANCER_NORMAL_LIKE_UP

LMOD1	SMID_BREAST_CANCER_NORMAL_LIKE_UP
LEPR	SMID_BREAST_CANCER_NORMAL_LIKE_UP
TGFBR3	SMID_BREAST_CANCER_NORMAL_LIKE_UP
TXNIP	SMID_BREAST_CANCER_NORMAL_LIKE_UP
LEF1	SMID_BREAST_CANCER_NORMAL_LIKE_UP
FOXN3	SMID_BREAST_CANCER_NORMAL_LIKE_UP
NDC80	PAM50
MKI67	PAM50
